# Geographical Variation in Body Size and Sexual Size Dimorphism in an Australian Lizard, Boulenger's Skink (*Morethia boulengeri*)

**DOI:** 10.1371/journal.pone.0109830

**Published:** 2014-10-22

**Authors:** Damian R. Michael, Sam C. Banks, Maxine P. Piggott, Ross B. Cunningham, Mason Crane, Christopher MacGregor, Lachlan McBurney, David B. Lindenmayer

**Affiliations:** Fenner School of Environment and Society, ARC Centre of Excellence for Environmental Decisions, and National Environment Research Program, The Australian National University, Canberra, Australia; Ecole Normale Supérieure de Lyon, France

## Abstract

Ecogeographical rules help explain spatial and temporal patterns in intraspecific body size. However, many of these rules, when applied to ectothermic organisms such as reptiles, are controversial and require further investigation. To explore factors that influence body size in reptiles, we performed a heuristic study to examine body size variation in an Australian lizard, Boulenger's Skink *Morethia boulengeri* from agricultural landscapes in southern New South Wales, south-eastern Australia. We collected tissue and morphological data on 337 adult lizards across a broad elevation and climate gradient. We used a model-selection procedure to determine if environmental or ecological variables best explained body size variation. We explored the relationship between morphology and phylogenetic structure before modeling candidate variables from four broad domains: (1) geography (latitude, longitude and elevation), (2) climate (temperature and rainfall), (3) habitat (vegetation type, number of logs and ground cover attributes), and (4) management (land use and grazing history). Broad phylogenetic structure was evident, but on a scale larger than our study area. Lizards were sexually dimorphic, whereby females had longer snout-vent length than males, providing support for the fecundity selection hypothesis. Body size variation in *M. boulengeri* was correlated with temperature and rainfall, a pattern consistent with larger individuals occupying cooler and more productive parts of the landscape. Climate change forecasts, which predict warmer temperature and increased aridity, may result in reduced lizard biomass and decoupling of trophic interactions with potential implications for community organization and ecosystem function.

## Introduction

Spatial and temporal variation in intraspecific body size is driven by differences in the heritability of phenotypic traits, and is the basis of evolution and adaptation to environmental change [1]–[3]. A substantial literature exists on the factors that influence body size, from which, reported patterns either support or contradict specific ecogeographical rules [4]–[7]. Bergmann's rule, for example, was one of the first ecogeographical generalizations to explain body size variation in endotherms. Bergmann's rule states that races (or as Bergmann probably intended, closely related species, especially congenerics) of warm-blooded vertebrates from cooler climates tend to be larger than races occupying warmer climates [8]. This rule, which is independent of gender, was originally reserved to explain interspecific differences in body size, although modern interpretations often extend the rule to include intraspecific investigations.

Bergmann's rule has received broad support in studies of birds [9], [10] and mammals [11]–[13]. However, studies on ectothermic vertebrates remain controversial [14]–[17]. For example, one study found that southern populations of the Australian frogs *Limnodynastes tasmaniensis* and *Litoria peronii* were significantly larger than northern populations [18]. Another study found that the mean body size of *Bufo bufo* in Europe decreased with latitude, but not altitude [19]. Similarly, other studies failed to find support for Bergmann's rule within anurans [9] or even within the Class Amphibia [20]. Studies on squamates also lack conclusive support for Bergmann's rule [21]. Instead, studies show a converse Bergmann cline or no obvious relationship between body size and latitude [14], [15], [22]–[25].

The lack of consensus among studies may be due to a paucity of investigations involving herpetofauna, particularly reptiles [6]. However, one reason why reptiles may not conform to Bergmann's rule may relate to the underlying mechanisms that drive the evolution of body size [7]. For example, the heat-conservation hypothesis predicts that larger animals are better at enduring cold temperatures because these animals need to produce less warmth in relation to their size to raise their temperature above that of their surroundings [8] - the classic physiological explanation for Bergmann's rule in endotherms. Because reptiles are unable to maintain a constant body temperature without using behavioral or physiological mechanisms, the heat-conservation hypothesis may not apply in this group [26]. Instead, mechanisms that relate to resource availability have been postulated as driving body size evolution in reptiles [7], [27]. For example, measures of primary productivity could impose a selective pressure on body size since body mass must be maintained by a sufficient food supply [28].

Clearly further research is required to test the mechanisms that drive body size evolution in reptiles, including whether observed patterns are consistent over different spatial scales [7]. Regional-scale studies, like the one we present here, are important because they provide information that may help develop rules that apply to organisms at a particular spatial scale. Furthermore, understanding spatial patterns in body size has broad implications for evaluating an organisms' response to environmental change [29]. With this in mind, we designed a heuristic study to address these knowledge gaps by examining body size variation in an Australian lizard, Boulenger's Skink *Morethia boulengeri*. Our primary objective was to determine if spatial patterns in body size and sexual size dimorphism existed in a widespread and common lizard species, but from a regional-scale, and if so, whether the observed variation could be explained by phylogenetic, environmental or ecological factors. We developed several hypotheses to explore these relationships further.

- Phylogenetic analysis can help to recognize new species. Because *M. boulengeri* occupies a large geographical area, the species may contain cryptic species with divergent phenotypic characteristics. Therefore, we investigated mitochondrial DNA divergence among samples from across our study area and a smaller number of samples from throughout the species' range. We predicted that body size variation may relate to an unknown phylogeny.
- Between-sex differences in body size are widespread among squamates [30], [31]. Snout-vent length (SVL) and head size are two traits which differ among sexes giving rise to the ‘fecundity selection’ hypothesis, whereby females evolve larger abdomens to accommodate more eggs, and the ‘sexual selection’ hypothesis whereby males' exhibit larger heads to facilitate male-male rivalry [32]. In suitable habitat, *M. boulengeri* can occur in high densities (421–1823 individuals/ha), including aggregations of mixed sex and age (D. Michael pers. obs.). These observations suggest that male-male rivalry is atypical in this species. Thus, we predicted that fecundity selection, as determined by SVL, would explain sexual differences in body size.
- Topography (elevation) is one component of a landscape that influences climatic conditions such as temperature and precipitation. Climatic factors are well documented in explaining body size variation in endotherms [12], [33], but lack conclusive support in squamate reptiles [14], [15], [25]. Thus, we predicted that body size will decrease with elevation, a trend consistent with studies on lizards.
- Environmental factors play an important role in influencing growth rate and body condition in reptiles [34]. Variation in morphological traits is mediated by food availability [35], anti-predatory behavior [36] and habitat fragmentation [37]. Moreover, a recent study found a positive relationship with *M. boulengeri* abundance and fallen timber [38], a critical resource in agricultural landscapes which provides both food and shelter. Thus, we predicted that habitat variables such as fallen timber and ground cover attributes might explain body size variation.

## Methods

### 1.1 Study area

**Figure 1:**
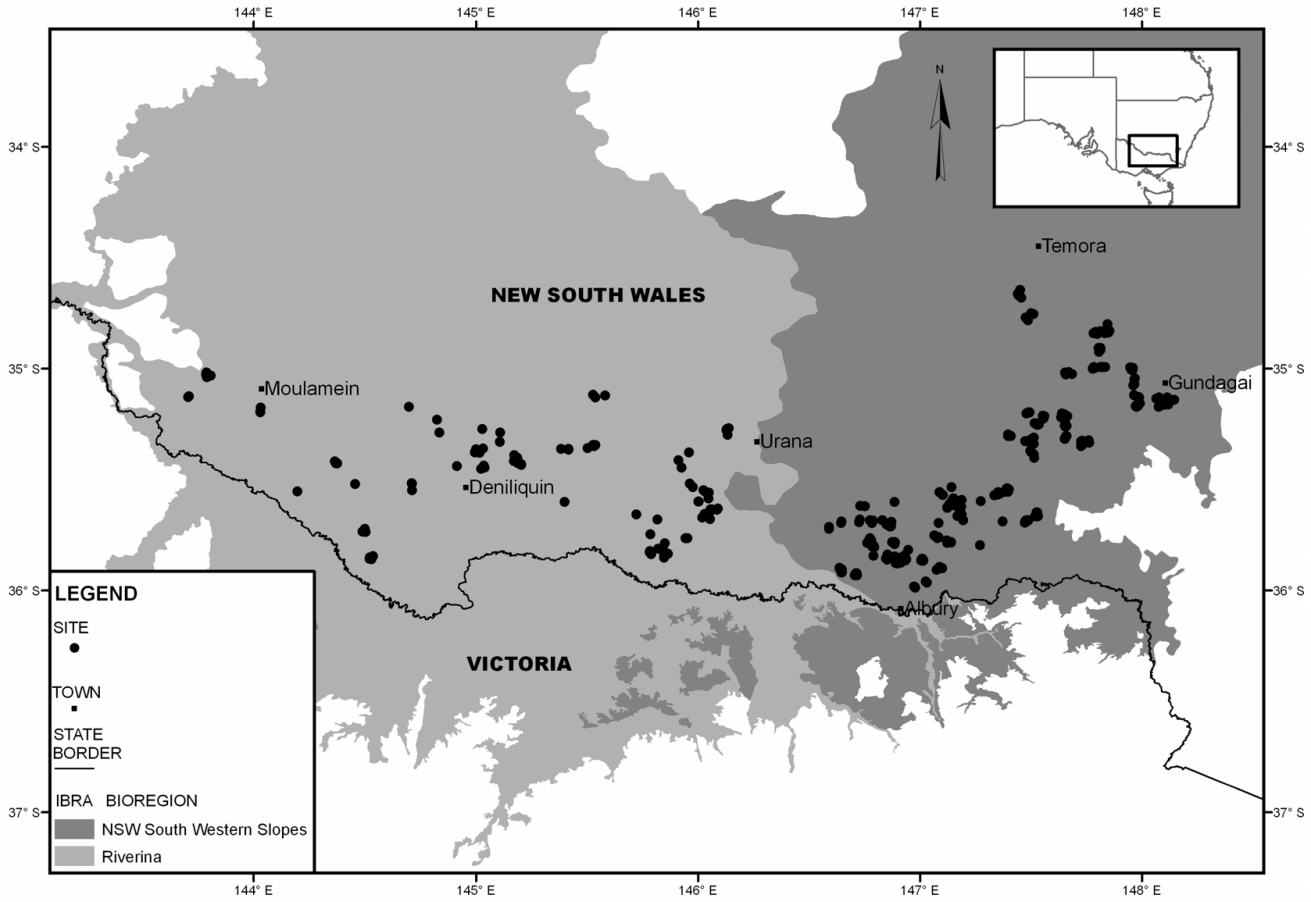
The Riverina and South-west Slopes study region in southern New South Wales, south-eastern Australia.

We conducted our study on private property in the temperate eucalypt woodlands of the Riverina and South-west Slopes (SWS) bioregions of southern New South Wales, Australia. This area is defined by Temora (34° 52′ 17″ S, 147° 35′ 06″ E) in the north, Albury (36° 04′ 44″ S, 146° 55′ 02″ E) in the south, Howlong in the east (35° 58′ 47″ S 146° 37′ 32″ E) and Moulamein (35° 01′ 59″ S 143° 43′ 42″ E) in the west (Figure 1). Our sampling sites were part of two broad-scale biodiversity monitoring programs on farming properties, travelling stock reserves and roadside verges [39]. The study area spans a 500 m elevation gradient from the plains in the west to the slopes in east. The native vegetation of the region has been extensively cleared and converted to broad-acre cropping and livestock production with remaining patches of native vegetation varying in size, shape and condition [40]. The most significant environmental changes driving patterns of reptile diversity in the region include habitat loss and modification, including extensive loss of native pasture and fallen timber [40]. Annual precipitation ranges from 320 mm in the Riverina to 900 mm in the SWS and is uniformly distributed throughout the year. January and February are the hottest months and the average minimum and maximum summer temperature ranges from 17°C–33°C in the Riverina to 18°C–32°C in the SWS. July is the coldest month and the average minimum and maximum winter temperature ranges from 0°C to 14°C [41].

### 1.2 Study animal

Boulenger's Skink *M. boulengeri* is a small (snout-vent length <55 mm), terrestrial heliotherm which occupies arid, semi-arid and temperate zones throughout central and south-eastern regions of Australia [42]. The species is oviparous, reaches sexual maturity in the first year and produces multiple clutches of variable size annually [43]. It is a habitat generalist commonly found basking on tree stumps and fallen timber, or sheltering beneath logs, rocks and leaf litter [40]. The species is one of the most abundant lizards in temperate Australia [38], [40], making it an ideal species for examining spatial patterns in body size.

### 1.3 Survey protocols

During September and October 2008, we actively searched for lizards along a 200 m×50 m transect (1 ha) on 369 sites (Figure 1). Clusters of 3–6 sites were nested within farm units (*n* = 98). We captured lizards by hand by turning over logs and rocks, raking through leaf litter and grass tussocks and visually scanning fallen timber for basking animals. We also inspected arrays of artificial refuges which we installed in June 2001 (SWS) and March 2008 (Riverina) as a reptile monitoring method [39]. We used digital calipers and digital scales (Satrue mini pocket scale, model: QM7241, Taiwan) to record SVL, total body length, head length (from the snout to the anterior margin of the occipital scale), head width (the widest distance between the jaws) and body mass (to nearest 0.1 g). We sexed individuals based on throat pigmentation (orange/red in breeding males and white in females) which is a valid technique for sexing this species during our survey period [43]. For approximately 30% of males sex was verified by hemipene eversion. We classified individuals as sub-adult or adult based on SVL at sexual maturity [43] (adult males >33 mm and adult females >37 mm). After all measurements were taken, we collected a tissue sample from the tail tip for genetic analysis.

### 1.4 Ethics statement

This study did not involve endangered or protected species and was carried out in strict accordance with the Australian National University Animal Care and Ethics Committee guidelines. Permission to access sites was granted by private landholders and the Livestock Health and Pest Authority under approval of the New South Wales National Parks and Wildlife Service (scientific license No. SL101022). The Murray Local Land Service is the primary contact for future access permission. All efforts were made to minimize stress during all animal handling procedures.

### 1.5 DNA extraction and sequencing

**Figure 2:**
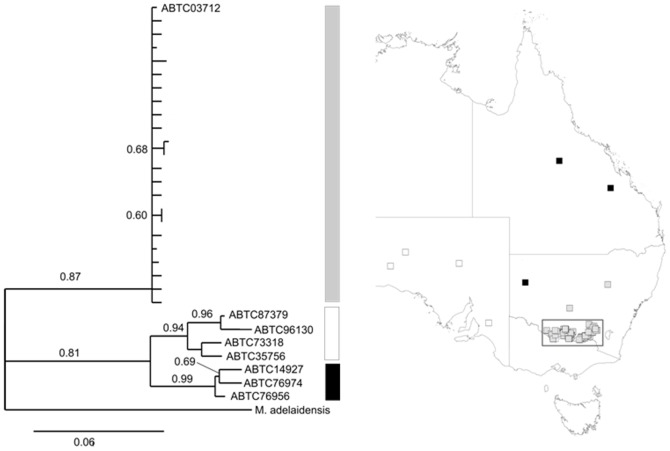
Maximum-likelihood phylogenetic tree. The tree represents 30 unique *Morethia boulengeri* haplotypes from 471 bp of mitochondrial DNA ND2 gene sequence, using *M. adelaidensis* (a closely-related species) as the outgroup. Sequences are coded according to major branches of the tree and their sampling locations are shown on the associated map. Sample names refer to the Australian Biological Tissue Collection numbers and unlabelled samples are those collected in this study (indicated by rectangle on map). Bootstrap values are shown on the figure and branches with less than 50% bootstrap support were collapsed.

We sequenced a 546 base pair fragment of the mitochondrial ND2 gene from 75 *M. boulengeri* sampled in this study (with at least one sample from each farm our sample size was adequate), nine *M. boulengeri* from other locations throughout the species' distribution in eastern Australia (from the Australian Biological Tissue Collection - see Figure 2 for collection numbers) and one Samphire Skink (*M. adelaidensis*), a closely-related species. We selected this gene because it is a common marker used to detect shallow phylogenetic structure [44]. We chose not to use faster evolving markers such as microsatellites as we were interested in testing for unrecognized species and not patterns of genetic differentiation caused by movement across the landscape. We extracted DNA according to the protocol of [44] and sequenced the samples as described in [45]. We edited and aligned the sequences using the program Geneious [46] and calculated haplotype diversity measures and a matrix of nucleotide differences between individuals in DnaSP [47]. We constructed a maximum likelihood phylogenetic tree with the PhyML program implemented in the Phylogeny.fr platform [48] using the HKY+G (gamma  = 0.327) substitution model, as selected according to AICc in (*imodeltest*) [49]. We performed a Mantel test in the R package Ecodist [50] to examine the correlation between the nucleotide difference matrix and Euclidean geographic distances.

### 1.6 Candidate variables

We used male and female adult SVL as a proxy for body size (because mass is confounded by reproductive status) and examined relationships with candidate explanatory variables collected at each site from four domains; 1) geography, 2) climate, 3) habitat and 4) management (see Appendix S1). First, we examined the relationship between SVL and geographical variables such as latitude and elevation, as these variables are widely used as climate surrogates [10], [11], [51]. Second, we examined climate variables such as maximum and minimum summer and winter mean temperature and precipitation values, as these are important in explaining body size in ectotherms [52]. We derived climate values from digital elevation models in ANUCLIM [53]. Last, we examined the relationship between body size and habitat, as well as management-related variables (see Appendix S1), as these factors influence food and retreat site availability [7].

### 1.7 Model construction and statistical analysis

We recorded a total of 761 individuals from 50% (185) of sites evenly distributed across the study area (i.e. absences were at the farm-level). The mean number of individuals per site was 4.11 (range 1–22 individuals). We collected morphological data from 424 individuals, representing 188 adult females, 149 adult males, 75 sub-adult females and 9 sub-adult males, of which 337 adults were used in the analysis. Neonates and juveniles (<6 months old) were not detected during the survey. Twenty-six females (13.83%) were gravid and included separately in the analysis. We used adjusted Wald statistics to examine differences in SVL and mass between males, gravid and non-gravid females [55]. Adjusted Wald statistics are assumed to have an F distribution and so observed Wald numbers are compared to critical values of an F distribution, on the relevant degrees of freedom. We determined significance at *P* = 0.05 if the Wald statistic was greater than four at one degree of freedom [55].

We used a statistical modeling approach to study relationships between lizard SVL (for both sexes separately) and candidate explanatory variables from the four domains; geography, climate, habitat and management. Because the variables were highly correlated, we dealt with co-linearity by modeling each variable separately and then selecting those variables from each domain with the highest level of significance. We constructed a parsimonious model by fitting statistically significant variables from each domain. As there were often multiple animals per site, we accounted for spatial autocorrelation at both the site and individual level by including *site* as a random effect and *sex* as a fixed effect along with the candidate variables. Our model selection procedure belongs to the general framework of general linear mixed models [54]. Statistical significance of an effect was assessed by calculating adjusted Wald statistics [55]. General model checking procedures were used to identify aberrant data and evaluate model assumptions. Statistical analyses were performed using GenStat Release 15.1 (VSN International, 2012).

## Results

### 2.1 Phylogenetic structure

We identified 29 mitochondrial ND2 gene haplotypes among the *M. boulengeri* sampled (haplotype diversity  = 0.749, SD = 0.047). The average number of nucleotide differences between samples was 5.32. Among the 75 sequences from samples collected in this study, there were 21 haplotypes with a haplotype diversity of 0.69 (SD = 0.054) and an average number of nucleotide differences between sequences of 1.48. There was a significant correlation between the nucleotide difference matrix and Euclidean geographic distances between individuals (Mantel *r* = 0.235, *P* = 0.001). The maximum likelihood phylogenetic tree revealed broad geographic phylogenetic structure in *M. boulengeri*, but on scale larger than our study region (Figure 2). We therefore chose not to include the phylogeny in subsequent models.

### 2.2 Sexual size dimorphism

**Figure 3:**
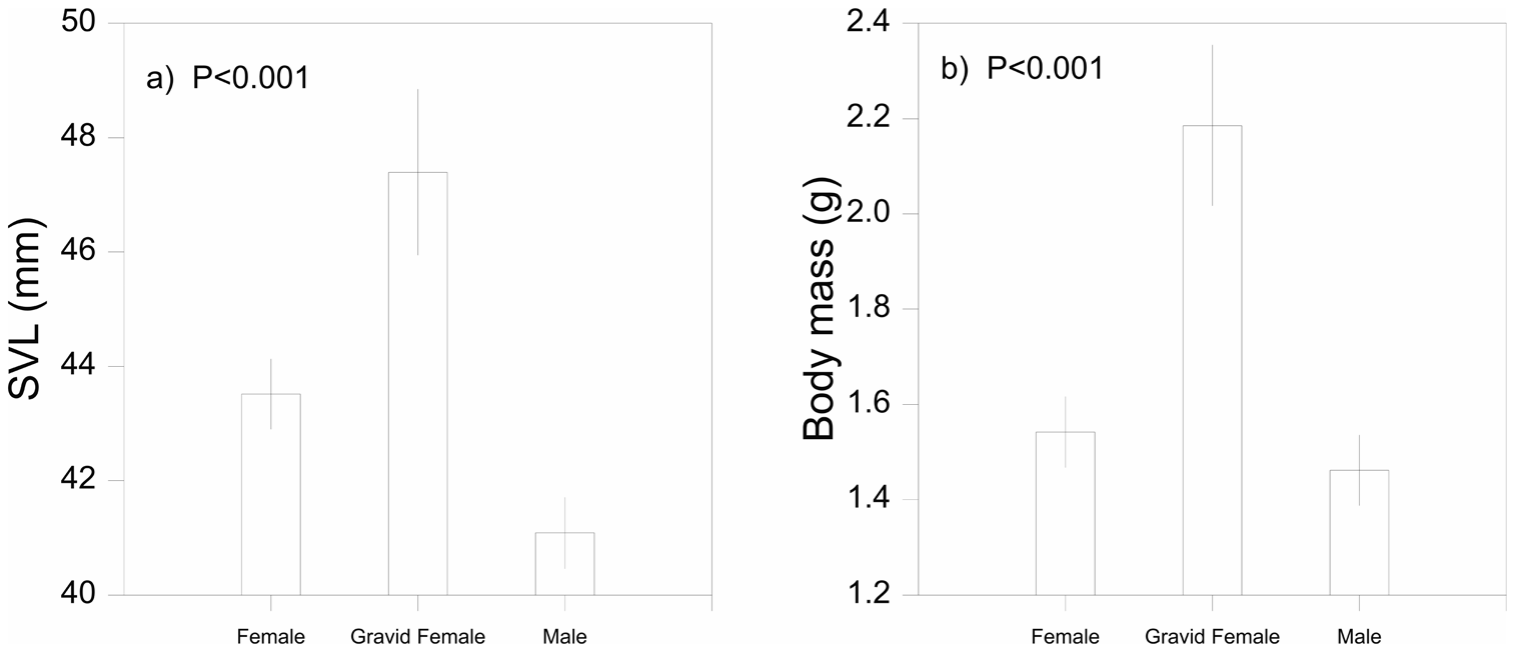
Sexual differences in body size. Regression analysis showing mean values and 95% confidence intervals for: a) snout-vent length (SVL mm), and b) body mass (g) between male, gravid female and non-gravid female *Morethia boulengeri* in southern New South Wales.

Linear regression analysis revealed several significant morphological differences between sexes (Figure 3). Gravid females (*N* = 26; SVL = 47.39±2.91 mm) had significantly longer SVL than non-gravid females (*N* = 155; SVL = 43.52±1.23 mm) and males (*N* = 147; SVL = 41.08±1.25 mm, *P*<0.001; Figure 3a). Non-gravid females had significantly longer SVL than males (*P*<0.001). Gravid females (*N* = 26; mass  = 2.18±0.33 g) had significantly greater body mass than non-gravid females (*N* = 155; mass  = 1.54±0.15 g), and males (*N* = 147; mass  = 1.46±0.15 g, *P*<0.001; Figure 3b). Non-gravid females were marginally heavier than males but this difference was not significant (*P* = 0.1). Head length (*P* = 0.6) and head width (*P* = 0.9) were also not significantly different between sexes.

### 2.3 Relationships between SVL and candidate variables

**Figure 4:**
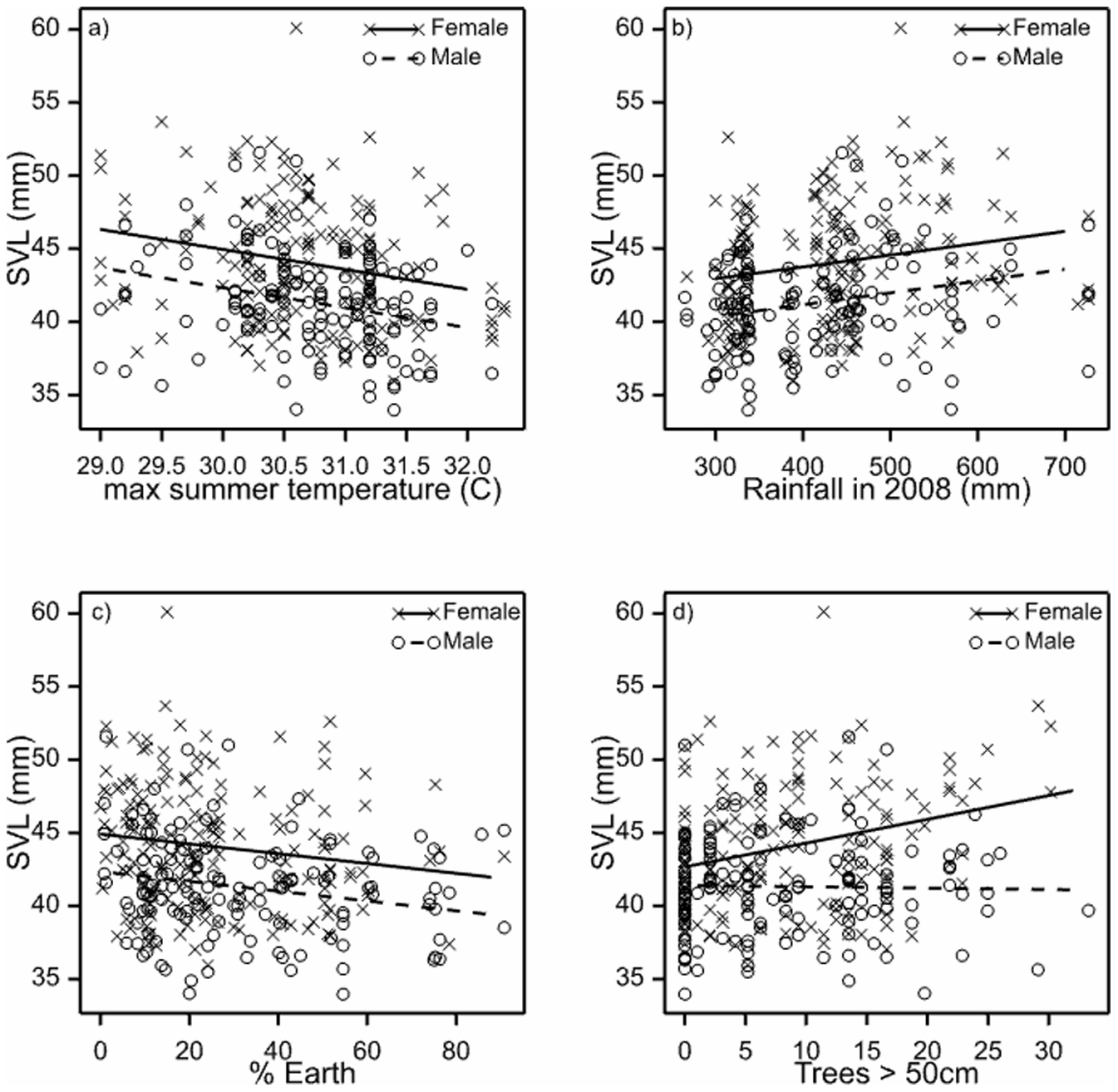
Regression analysis of *Morethia boulengeri* and candidate variables. The relationships show a positive association with snout-vent length (SVL) and: (a) temperature, (b) rainfall (c) percent bare ground cover, and (d) density of mature trees >50 cm diameter.

We found no significant relationship between male or female SVL and any of the geographical variables (e.g. bioregion, latitude or elevation). In contrast, all of the climate variables were important in explaining male and female SVL variation. However, these variables were highly correlated, so we selected mean maximum summer temperature (*P*<0.001; Figure 4a) and mean rainfall in 2008 (*P*<0.001; Figure 4b) based on their significance levels to use in the final model. Our habitat models included percent cover of bare ground (*P* = 0.003; Figure 4c) and the density of mature trees (*P* = 0.01; Figure 4d). Furthermore, we found a significant interaction between sex and the density of mature trees (*P* = 0.002), whereby female SVL increased relative to the density of mature trees. In relation to management models, we found no significant relationship with SVL and any of the candidate variables from this domain. In the final model, both climate variables remained significant, whereby SVL increased relative to decreasing temperature and increasing rainfall. However, in the presence of the climate variables, bare ground and large trees were no longer significant.

## Discussion

We examined factors affecting body size variation in a widespread Australian lizard on a regional-scale. Our key findings include: 1) Very limited genetic divergence among sampled *M. boulengeri* suggesting body size variation was not due to unrecognized species (Figure 2), 2) Strong evidence of sexual size dimorphism in SVL, especially in respect to gravid females, lending support for the fecundity selection hypothesis (Figure 3), and 3) Climate variables, rather than ecological, geographical or land management variables, were the best candidates for explaining differences in overall body size (SVL) in this system (Figure 4). We further discuss these findings in the remainder of the paper and conclude with some key implications for biodiversity conservation in changing landscapes.

### 3.1 Phylogenetic structure

Our analysis revealed broad geographic phylogenetic structure in *M. boulengeri* but on scale larger than our study region (Figure 2). Because we were primarily interested in testing for cryptic species within our study area, we chose not to use faster evolving markers such as microsatellites. Microsatellites are commonly used to explore patterns of divergence caused by movement across the landscape [44]. Therefore, we are confident that body size variation in *M. boulengeri* was not due to a previously unrecognized subspecies or species complex within our sample.

### 3.2 Sexual size dimorphism

Our data suggests that sexual size dimorphism in *M. boulengeri* was influenced by fecundity selection. Sexual differences in body size and shape are among the most common expressions of sexual dimorphism in lizards [32]. We found *M. boulengeri* was sexually dimorphic in SVL (Figure 3a) and to some degree, body mass (Figure 3b). By contrast, *M. boulengeri* was not sexually dimorphic in head length or head width. Our surveys were conducted during the species breeding season [43] and hence, sexual differences in body mass were probably attributed to embryo development in gravid females (Figure 3b). In species where offspring number is not genetically determined, there is a correlation between offspring number and female body size [56], a relationship known as the fecundity selection hypothesis. This hypothesis predicts that females will evolve larger abdomen length to accommodate more eggs. The more offspring a species produces can be a measure of fitness and contribute to divergent morphological differences such as variation in SVL [32]. In contrast, sexual differences in head size are attributed to male-male rivalry and arise through sexual selection processes. Our findings clearly show that gravid females had larger SVL than non-gravid females (which were possibly sexually immature or first year breeders) and males, suggesting that the fecundity selection hypothesis, rather than the sexual selection hypothesis, is a plausible explanation for sexual size dimorphism in this species. However, further studies are required to further test this assumption.

### 3.3 Correlates of body size variation

Our data suggests that body size variation (SVL) in *M. boulengeri* was consistent with a Bergmann cline. However, other factors aside from latitude were important in this study. For example, our exploratory analysis showed SVL was not related to any geographical variables (including latitude) but instead was related to both climate and ecological variables (Figure 4). Both temperature and precipitation were related to body size variation, whereby male and female SVL increased as a function of decreasing temperature and increasing rainfall (Figure 4a, b). Furthermore, we found SVL decreased as the percent cover of bare ground increased (Figure 4c), and female SVL increased relative to the increase in the density of mature trees (Figure 4d). However, when we included the two climate variables in the final model, the two habitat variables no longer remained significant. Instead, we found temperature and rainfall were the best two candidates for explaining body size variation in this system, a trend congruent with other studies that use climate variables as a proxy for latitude when investigating Bergmann clines [57]. Our findings thus contradict with our original hypothesis that lizard body size decreases with elevation and contrasts with other studies on squamates that have investigated the relationship between climate variables and body size variation [14]–[16].

One physiological explanation for Bergmann's rule in endotherms is the heat-conservation hypothesis, which states that larger animals are better at enduring colder climates than smaller animals [8]. This hypothesis, may also apply to some ectotherms, especially organisms that are capable of behaviorally regulating their body temperature [58]. Many lizards are capable of maintaining a preferred body temperature by moving between sun and shade (shuttling) or changing their body posture [26], in which case, the heat-conservation hypothesis may apply in shuttling heliotherms such as *M. boulengeri*, which spends large amounts of time alternating between sun and shade. In cooler climates, larger lizards may have a selective advantage over smaller individuals due to their ability to retain heat for longer, a hypothesis that requires further investigation. Alternatively, body size differences may be driven by measures of productivity, including resource availability [35]. However, examining prey relationships was beyond the scope of this study and is an area of research that requires further investigation.

### 3.4 Implications for management

For many endotherms, it is well established that on geological timescales (over millennia), body size decreases as a function of increasing temperature [9]–[12]. But how adaptive is this trait and does it apply to populations over contemporary timescales (e.g. several decades)? Recent studies have shown that body size of birds and mammals are declining due to rising global temperatures [2], [59], a phenomenon reported to be the third universal response of climate warming [29]. The implications of climate-induced declines in body size are not fully understood, but are likely to effect trophic interactions, community organization and ecosystem function [60]. From an anthropocentric perspective, agricultural sustainability is dependent on a functioning ecosystem to provide environmental services such as pollination and pest control [61]. Common lizard species such as *M. boulengeri* play pivotal roles in maintaining ecosystem function as they not only prey on a wide range of herbivorous invertebrates [40], but they also contribute to overall food web dynamics.

In temperate Australia, *M. boulengeri* is preyed upon by a wide variety of species, including several small threatened elapids [40] and birds such as the Bush Stone Curlew *Burhinus grallarius* [62]. Climate change forecasts, which predict warmer temperature, decreased precipitation and increased aridity in south-eastern Australia [63], could potentially result in reduced lizard biomass, decoupling of trophic interactions and increased stress on reptile populations due to compositional changes to vegetation communities and ecosystem shifts [64]. Management practices that ultimately improve the quality and quantity of native vegetation in agricultural landscapes will not only improve biodiversity outcomes for common lizard species such as *M. boulengeri* [65], but may also mediate evolutionary divergent processes, such as changes in body size, that are predicted by habitat fragmentation studies [37].

## Supporting Information

Appendix S1 **Candidate variables used in regression models.**
(DOC)
