# Supplementary material for: Geographical Variation in Body Size and Sexual Size Dimorphism in an Australian Lizard, Boulenger's Skink (Morethia boulengeri)
Source: PLoS One. 2014 Oct 22;9(10):e109830. doi: 10.1371/journal.pone.0109830 (PMC4206273; doi:10.1371/journal.pone.0109830)
Supplement: Appendix S1 — Candidate variables used in regression models. (DOC) [file pone.0109830.s001.doc]

**Appendix S1.** Candidate explanatory variables associated with four broad domains used in the generalised linear regression model-selection process.

| **Domain** | **Variable** | **Description** |
| --- | --- | --- |
| Geography | Bioregion | Sites were categorized according to their location within one of two IBRA bioregions: Riverina (*N* = 111 sites) or South-west Slopes Bioregion (*N* = 258 sites) |
|  | District | Sites were assigned to a district category based on their location to one of 20 nearest towns. |
|  | Latitude | Derived from GPS (Garmen Etrax™) |
|  | Longitude | Derived from GPS (Garmen Etrax™) |
|  | Elevation (m) | Derived from Digital Elevation Models (DEM) |
| Climate | Summer maximum temperature (ºC) | Derived from DEM |
|  | Winter maximum temperature (ºC) | Derived from DEM |
|  | Summer minimum temperature (ºC) | Derived from DEM |
|  | Winter minimum temperature (ºC) | Derived from DEM |
|  | Precipitation in 2007 (mm) | Mean precipitation during the period 1 January – 31 December |
|  | Precipitation in 2008 (mm) | Mean precipitation during the period 1 January – 31 December |
| Habitat | Vegetation type | Eight categories based on Keith’s vegetation Classification |
|  | Number of native overstorey stems | The total number of tree stems averaged over three 20 x 20 m plots along a 200 m transect |
|  | Density of large trees (No./ha) | The number of trees > 50 cm diameter at breast height counted over 1 ha (50 m x 200 m) |
|  | Density of logs (No./ha) | The number of logs > 10 cm diameter and > 1 m in length averaged over three 20 x 20 m plots along a 200 m transect, adjusted to 1 ha. |
|  | Percent cover of native herbs, native grass, exotic grass, exotic broad-leaf plants, bare ground, leaf litter and rock | Ground cover abundance estimates averaged over twelve 1 m x 1 m plots along a 200 m transect |
| Management | Land use | Five land use categories based on interviews with landholders: 1) sites used for livestock production, 2) sites excluded from livestock grazing pre-2000, 3) sites excluded from livestock grazing in 2007, 4) travelling stock reserves, and 5) linear or block tree plantings |
|  | Grazing pressure | The sum of binary scores collected over three 20 m x 20 m plots representing 0 - no evidence of grazing, or 1 - evidence of native herbivores and/or livestock grazing |
